# Birth month and risk of skin tumors—Follow up of six million Caucasians born from 1950 to 2014 in Sweden

**DOI:** 10.1002/cam4.3286

**Published:** 2020-07-06

**Authors:** Rickard Ljung, Mats Talbäck, Amal R. Khanolkar, Maria Feychting

**Affiliations:** ^1^ Unit of Epidemiology Institute of Environmental Medicine Karolinska Institutet Stockholm Sweden; ^2^ GOS Institute of Child Health University College London London UK

**Keywords:** birth month, childhood, melanoma, skin cancer, sun exposure, ultraviolet radiation

## Abstract

**Background:**

Some studies hypothesize that birth month—as a proxy of exposure to ultraviolet radiation in early infancy—is associated with increased risk of skin tumors.

**Methods:**

We studied a national cohort of all 5 874 607 individuals born in Sweden to parents of Swedish or Nordic origin as a proxy for Caucasian origin, 1950 to 2014. The cohort was followed for incident skin tumors, including squamous cell carcinomas and melanomas but not basal cell carcinomas, through 2015 from birth up to age 65 for the oldest cohort. Cox regression estimated the association between month of birth and risk of skin tumors in models adjusted for sex, calendar period, and education. Crude observed to expected ratios were also calculated.

**Results:**

There were 33 914 cases of skin tumors, of these, 3025 were squamous cell cancer, 16 968 malignant melanoma and 8493 melanoma in situ/other and 5 428 squamous cell in situ/other in 192 840 593 person‐years of follow‐up. Observed to expected ratios by month of birth showed no association between month of birth and risk of skin tumors, and the same result was seen when Cox regression analysis was used. Subgroup analyses by sex, educational level, calendar period, or age at follow‐up similarly showed no association.

**Conclusion:**

This large register‐based cohort study showed no evidence of a higher risk of skin tumors in those born during the spring. Thus, this study lends no support to the hypothesis that birth during spring is a major risk factor for later skin tumors.

## Graphical abstract

A study of six million persons born in Sweden from 1950 to 2014 lends no support to the hypothesis that birth during spring is a major risk factor for later skin tumors.

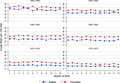

## 1 INTRODUCTION

Exposure to ultraviolet radiation during childhood is an important known risk factor for skin tumor, especially malignant melanoma, and has been shown in some but not all studies.^1^, ^2^, ^3^, ^4^ However, there is a paucity of data on the importance of exposure to ultraviolet radiation during early infancy. The few studies addressing this issue show conflicting results.^5^, ^6^, ^7^, ^8^, ^9^ It has been hypothesized that infants born during spring have a higher risk of skin tumors as they are exposed to ultraviolet radiation in a susceptible period compared to those born later in the year. However, the basis for the hypothesis and the methodological soundness of some of these studies has been questioned.^5^, ^9^, ^10^, ^11^ Also, as month of birth is not evenly distributed in the population it is of great importance to have full control of the population at risk.

We aimed to study the association between birth month and skin tumor in a cohort restricted to Caucasians in a national setting with high quality registers and completes coverage of person‐time at risk, cancer occurrence and follow‐up.

## 2 MATERIAL AND METHODS

### 2.1 Study design

We conducted a population‐based cohort study of all individuals born in Sweden 1950 to 2014 (N = 7 245 453). Restricting the cohort to those born in Sweden to Swedish born parents or those born in neighboring Nordic countries helped ensure that individuals were most likely of Caucasian origin (N = 5 874 607). Information on date of birth, date of death and date of emigration was obtained from the Total Population Register.^12^ Occurrence of first skin tumor was obtained from the Swedish Cancer Register.^13^ We used the Multi‐Generation Register to identify the country of birth of the parents. Registers were linked using the unique personal identity number assigned to all Swedish residents. Subjects were followed until first diagnosis of skin tumor and censored on migration, death or end of study period (31 December 2015).

### 2.2 Exposure

Date of birth for all 5.9 million individuals was categorized into birth month with March as the reference. Also, summer (June, July, August) was reference to spring (March, April, May), autumn (September, October, November), and winter (December, January, February) in the analyses of season of birth.

### 2.3 Outcome—Skin tumor

Registration of all new primary malignancies is statutory in Sweden and the completeness of the Cancer Register is estimated to be high.^13^ Skin tumor diagnoses were defined using the International Statistical Classification of Diseases and Related Health Problems, Seventh Revision (ICD‐7), code 190‐191, corresponding to C43‐C44 in ICD‐10. The ICD‐7 revision was used because it was the only ICD version available in the Swedish Cancer register from 1960 throughout the whole study period. We included squamous cell carcinomas and melanomas, here after referred to as skin cancer/tumor. Basal cell carcinomas, despite being the by far most common cancer type, was excluded as it has only been routinely collected in the cancer register from 2004 and onwards in Sweden.

### 2.4 Covariates

The year of birth was categorized in 5‐year calendar periods, 1950‐1954, 1955‐1959, 1960‐1964, 1965‐1969, 1970‐1974, 1975‐1979, 1980‐1984, 1985‐1989, 1990‐1994, 1995‐1999, 2000‐2004, 2005‐2009, 2010‐2014. Information on educational level at age 30 was ascertained from the Longitudinal Integration Database for Health Insurance and Labour Market Studies, held by Statistics Sweden. Highest attained educational level was categorized as: primary (ie less than 10 years of education), secondary (ie 10‐12 years of education), and tertiary (ie ≥13 years of education, corresponding to university education).

### 2.5 Statistical analyses

We calculated age‐adjusted incidence rates by birth month for men and women separately, stratified by calendar period and by age at follow‐up. The distribution of birth month among skin tumor cases was compared to percentages of births per month and by percentages of person‐time at risk in the general population by birth months as ratios of observed to expected cases. Also, we estimated hazard ratios (HR) of skin tumor between strata of birth month by multivariable Cox regression, using age as timescale with 95% confidence intervals (95% CIs). Analyses were adjusted for sex and calendar year and additionally for education level where applicable. In sensitivity analyses we restricted the outcome to malignant melanoma cases only. Also, in a secondary analysis we used the same time period, reference (fall) and inclusion criteria as Crump et al^6^ and compared the results from a multivariable Cox regression with the results of logistic regression, as used in Crump et al^6^ analyzing those born between 1973 to 2008, including 3 795 592 subjects, and 1681 cases of skin tumor. Statistical analyses were conducted using STATA version 14.

## 3 RESULTS

The study population consisted of 5 874 607 individuals, including 3 018 521 men (51.4%) and 2 856 086 women (48.6%) yielding 99 032 088 and 93 0808 505 person‐years of follow‐up, respectively. Characteristics of the 33 914 skin tumor cases are shown in Table 1. Of the 33 914 cases of skin tumors, 3025 were squamous cell cancer, 16 968 malignant melanoma, 8493 melanoma in situ/other and 5428 squamous cell in situ/other. Incidence was higher in women and increased by age.

**TABLE 1:** Characteristics of 33 914 skin tumor, and of which 25 597 malignant melanoma, in individuals born from 1950 to 2014, followed until 31 December 2015, in Sweden

Birth year	All skin tumors	Women	Melanoma	Women	Age at diagnosis			
	N	(%)	N	(%)	Mean	(SD)	Min	Max
1950‐1954	9625	53.4	6073	53.5	54.1	9.5	6.7	65.9
1955‐1959	7095	55.4	4938	55.9	49.3	9.0	6.5	60.9
1960‐1964	5501	59.9	4268	60.9	44.7	8.3	1.5	55.8
1965‐1969	4722	61.9	3970	61.8	40.6	7.5	0.3	50.8
1970‐1974	3134	62.5	2790	63.1	36.2	6.8	1.3	45.6
1975‐1979	1822	64.2	1676	64.9	32.5	5.7	4.5	40.8
1980‐1984	968	65.4	912	65.2	28.9	4.3	7.0	35.6
1985‐1989	658	68.2	624	68.1	24.9	3.4	10.3	30.7
1990‐1994	287	66.6	260	66.2	20.6	2.8	3.2	25.7
1995‐1999	81	54.3	70	57.1	17.0	2.9	2.9	20.8
2000‐2004	15	73.3	11	72.7	9.5	4.3	0.7	14.7
2005‐2009	5	^a^	5	^a^	6.4	2.3	3.3	8.8
2010‐2014	^a^	^a^	^a^	^a^	^a^	^a^	^a^	^a^

^a^ Three cases or less.

A crude comparison of observed to expected ratios of skin tumor cases by birth month showed the highest estimates in April for men and March for women, and the lowest in September for men and November for women. (Table 2).

**TABLE 2:** Observed to expected ratio of skin tumor cases by month of birth. Expected cases are calculated for both the distribution of total person‐time and for the distribution of individuals born and alive at any time during follow‐up. Proportion of person‐time and births are calculated for men and women combined. Those born 1950 to 2014, followed until 31 December 2015, in Sweden

Month	Observed		Expected by proportion person‐years			Expected by proportion individuals born			
	Observed cases	Proportion cases	Proportion years	Expected	Obs/Exp 95%CI	Individuals born	Proportion individuals	Expected	Obs/Exp 95% CI
*Men*									
Jan	1206	8.51%	8.29%	1174.5	1.03 (0.97‐1.09)	249 126	8.24%	1167.4	1.03 (0.97‐1.09)
Feb	1161	8.19%	8.13%	1152.1	1.01 (0.95‐1.07)	244 316	8.09%	1146.0	1.01 (0.95‐1.07)
Mar	1340	9.46%	9.63%	1365.0	0.98 (0.93‐1.04)	287 190	9.48%	1342.5	1.00 (0.94‐1.05)
Apr	1461	10.31%	9.60%	1359.7	1.07 (1.02‐1.13)	285 449	9.44%	1337.5	1.09 (1.04‐1.15)
May	1371	9.68%	9.28%	1314.5	1.04 (0.99‐1.10)	276 885	9.18%	1301.3	1.05 (1.00‐1.11)
Jun	1160	8.19%	8.44%	1195.9	0.97 (0.91‐1.03)	256 242	8.50%	1203.7	0.96 (0.91‐1.02)
Jul	1139	8.04%	8.38%	1188.0	0.96 (0.90‐1.02)	258 376	8.55%	1211.6	0.94 (0.89‐1.00)
Aug	1115	7.87%	8.05%	1141.0	0.98 (0.92‐1.04)	248 305	8.26%	1169.8	0.95 (0.90‐1.01)
Sep	1082	7.64%	8.08%	1144.5	0.95 (0.89‐1.00)	246 029	8.15%	1154.5	0.94 (0.88‐1.00)
Oct	1082	7.64%	7.74%	1096.6	0.99 (0.93‐1.05)	234 281	7.80%	1105.1	0.98 (0.92‐1.04)
Nov	971	6.85%	7.12%	1008.8	0.96 (0.90‐1.03)	214 536	7.12%	1009.0	0.96 (0.90‐1.03)
Dec	1081	7.63%	7.26%	1028.5	1.05 (0.99‐1.12)	217 786	7.20%	1020.6	1.06 (1.00‐1.13)
*Women*									
Jan	1622	8.21%	8.29%	1636.7	0.99 (0.94‐1.04)	234 874	8.24%	1626.8	1.00 (0.95‐1.05)
Feb	1601	8.11%	8.13%	1605.6	1.00 (0.95‐1.05)	230 816	8.09%	1597.0	1.00 (0.95‐1.05)
Mar	1984	10.05%	9.63%	1902.1	1.04 (1.00‐1.09)	269 441	9.48%	1870.9	1.06 (1.01‐1.11)
Apr	1896	9.60%	9.60%	1894.9	1.00 (0.96‐1.05)	269 092	9.44%	1863.9	1.02 (0.97‐1.07)
May	1861	9.43%	9.28%	1831.8	1.02 (0.97‐1.06)	262 647	9.18%	1813.4	1.03 (0.98‐1.07)
Jun	1678	8.50%	8.44%	1666.5	1.01 (0.96‐1.06)	242 818	8.50%	1677.4	1.00 (0.95‐1.05)
Jul	1629	8.25%	8.38%	1655.5	0.98 (0.94‐1.03)	243 976	8.55%	1688.4	0.96 (0.92‐1.01)
Aug	1537	7.78%	8.05%	1590.0	0.97 (0.92‐1.02)	236 708	8.26%	1630.2	0.94 (0.90‐0.99)
Sep	1559	7.90%	8.08%	1594.9	0.98 (0.93‐1.03)	232 648	8.15%	1608.9	0.97 (0.92‐1.02)
Oct	1538	7.79%	7.74%	1528.2	1.01 (0.96‐1.06)	223 898	7.80%	1540.0	1.00 (0.95‐1.05)
Nov	1384	7.01%	7.12%	1405.8	0.98 (0.93‐1.04)	203 820	7.12%	1406.1	0.98 (0.93‐1.04)
Dec	1456	7.37%	7.26%	1433.2	1.02 (0.96‐1.07)	205 348	7.20%	1422.2	1.02 (0.97‐1.08)

**FIGURE 1:**
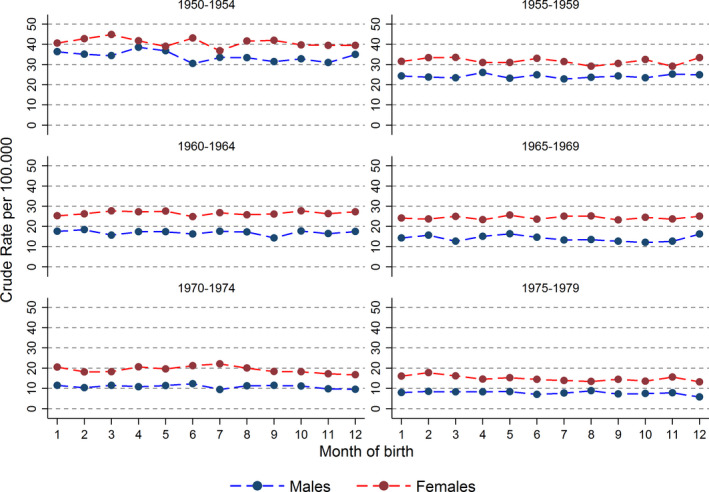
Crude rate of skin tumor by month of birth stratified by calendar birth period. Those born 1950‐2014

**FIGURE 2:**
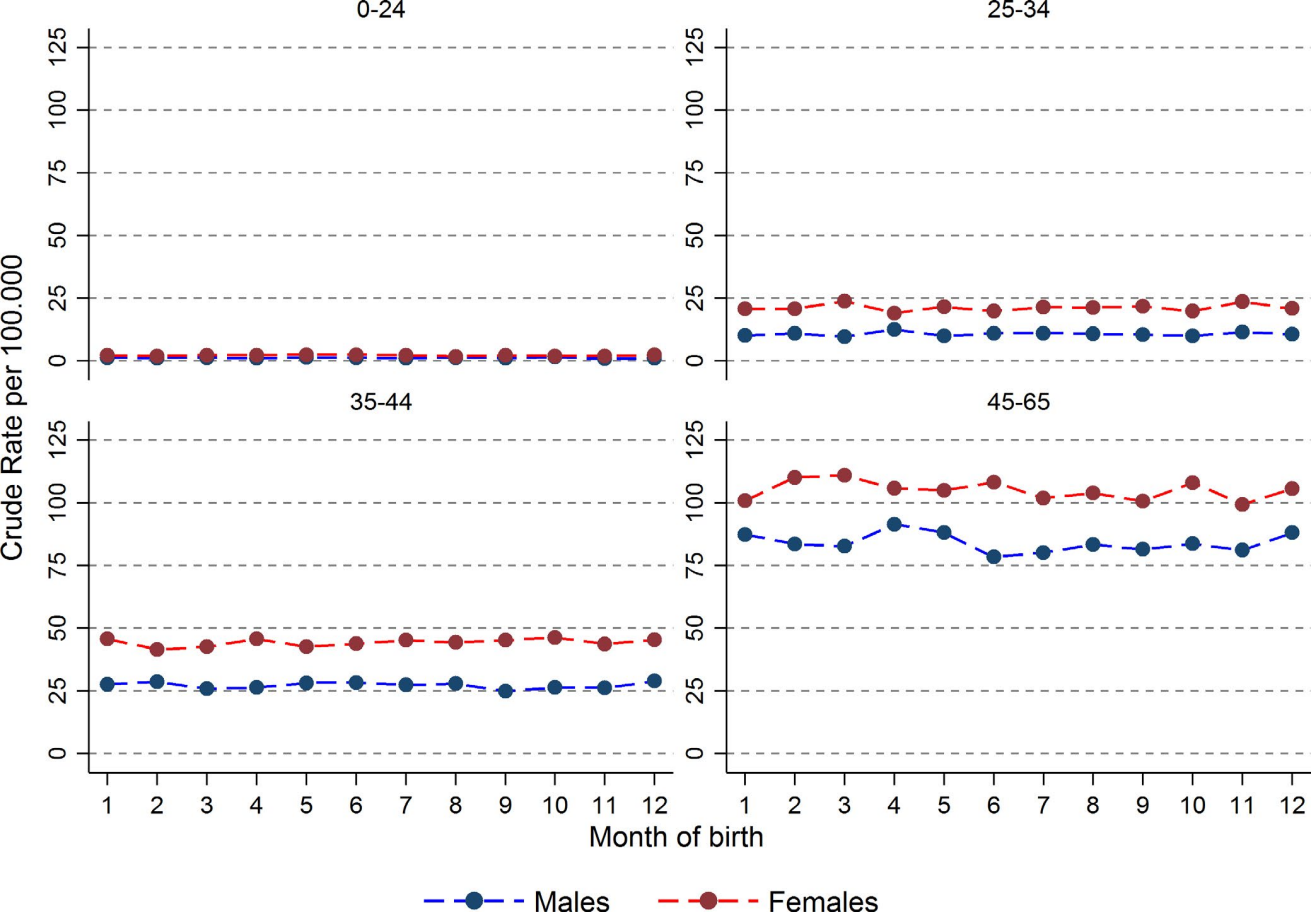
Crude rate of skin tumor by month of birth stratified by age at follow up. Those born 1950‐2014

Incidence of skin tumor by sex stratified by calendar birth period, presented by the first five years within each 10‐year calendar birth period, or age at follow‐up showed no difference between months of birth (Figures 1 and 2). Subgroup analyses by sex, educational level, calendar period, or age at follow‐up similarly showed no association (data not shown).

There was no association between month or season of birth and risk of skin tumor in the regression analyses. (Table 3). Also, restricting to malignant melanoma as outcome (N = 16 968) yielded no association with birth month (*P* = .682) (not shown). Mimicking the previous analyses on Swedish data by Crump et al,^6^ using same time period, reference (fall) and inclusion criteria on our data yielded a HR of 1.10 (0.96‐1.26) for Spring compared to an odds ratio of 1.20 (1.05‐1.37).

**TABLE 3:** Incidence rate ratios and 95% confidence intervals for month of birth and season and risk of skin tumor in individuals born 1950‐2014 in Sweden, ages 0‐65 and followed for skin tumor from birth until 31 December 2015

Month	Crude	Adjusted for sex and calendar birth year
	IRR, 95% CI	IRR, 95% CI
Jan	0.98 (0.94,1.03)	0.98 (0.93,1.03)
Feb	0.99 (0.94,1.04)	0.99 (0.94,1.04)
Mar (ref)	1	1
Apr	1.02 (0.97,1.07)	1.02 (0.97,1.07)
May	1.01 (0.96,1.06)	1.01 (0.97,1.06)
Jun	0.99 (0.94,1.04)	1.00 (0.95,1.05)
Jul	0.98 (0.93,1.03)	0.99 (0.94,1.04)
Aug	0.99 (0.94,1.05)	1.00 (0.95,1.05)
Sep	0.98 (0.93,1.03)	0.99 (0.94,1.04)
Oct	1.01 (0.96,1.06)	1.02 (0.97,1.08)
Nov	0.98 (0.93,1.03)	1.00 (0.95,1.05)
Dec	1.03 (0.97,1.08)	1.05 (1.00,1.11)
Season		
Winter	1.01 (0.98,1.04)	1.01 (0.98,1.04)
Spring	1.02 (0.99,1.05)	1.01 (0.98,1.04)
Summer (ref)	1	1
Autumn	1.00 (0.97,1.03)	1.01 (0.98,1.04)
		

## 4 DISCUSSION

This large national register‐based study of 5.9 million individuals of Caucasian origin born from 1950 to 2014 showed no association between birth month and later risk of skin tumors. Our negative findings support a similarly large study from Germany showing no association.^9^ However, in contrast, three previous studies found an association between month of birth and malignant melanoma.^5^, ^6^, ^7^

The main strengths of this study include the total population‐based cohort design, the large sample size, the complete nationwide coverage of the study exposures (birth month), outcome (skin tumor) and covariates and complete follow‐up using valid national Swedish registers with negligible missing data. Also, restricting the study population to those born in Sweden to Nordic born parents ensured that the vast majority of the study population was of Caucasian origin known to have a higher risk of skin tumors.^14^, ^15^ A limitation could be that we do not know if parents with high health awareness, including use of sun‐protection, chose not to have children born during seasons of high sun exposure (spring and summer). However, we think this is of minor importance, also it is not so easy to plan a pregnancy and date of delivery.

There is an on‐going debate on the most appropriate methodology to test the hypothesis that birth month is associated with increased risk of skin tumors in adulthood.^5^, ^6^, ^7^, ^8^, ^9^, ^10^, ^11^ It is essential to allocate the correct person‐time at risk by incorporating information on the distribution of birth month in the general population. In Sweden, the spring months have the highest number of births although this seasonal pattern of birth has attenuated in recent decades. We used birth month available for all subjects as the exposure and in a regression analyzed the estimates for each month with March as the reference. Hence, we have adequately categorized the exposure for all individuals.

Previous studies that reported an association were considerably smaller: 210, 1745 and 1595, melanoma cases, respectively.^5^, ^6^, ^7^ One of these studies, also from Sweden, partly overlaps with our study population.^6^ This study included all persons born from 1973 and onwards, whereas we studied those born between 1950 and 2014. They found an association between spring birth and cutaneous malignant melanoma (OR 1.21, 95% CI 1.05‐1.39, reference fall) in the very young (maximum age 37). However, restricting our analyses to the criteria used in Crump et al^6^ yielded a HR of 1.10 (0.96‐1.26) for Spring using Cox regression compared to an odds ratio of 1.20 (1.05‐1.37) using logistic regression as done by Crump et al. The odds ratio using our data is very close to the one presented by Crump et al,^6^ hence we argue that the different interpretations of the findings may in part be due to the statistical model used for analyses. We used Cox regression to account for person time at risk.

The negative findings in a large German study analyzed 28 374 melanoma cases of all ages.^8^, ^9^ The most appropriate age spans to study remain unclear. One could argue that early exposure to ultraviolet radiation should induce cancer to develop in young adulthood. Sun exposure during childhood has been shown to increase the risk for later skin tumors.^3^ However, skin tumors are rare in young adults making results susceptible to random variation.

The hypothesis tested is the assumption that infants born in spring are more exposed to strong ultraviolet radiation in a susceptible period of life. In Sweden there are guidelines explicitly recommending avoidance of direct sun light for children below one year of age.^16^ Thus, most parents are probably very reluctant to expose their newborn infants to direct sun. However, in other parts of the world intentional direct sun exposure in infancy is a concern.^17^, ^18^, ^19^ Sunbathing in Sweden has increased over the last decades with a large part of the population repeatedly exposed to strong ultraviolet radiation during holidays in Southern Europe, South East Asia and Northern Africa. The incidence of malignant melanoma is increasing steadily as observed in our study and with a graded manner (with increasing HR) for later birth cohorts compared to the earliest birth cohort born in 1960 in this study. Thus, the increase in exposure to ultraviolet radiation in childhood, adolescence and adulthood, unrelated to birth month and exposure in very early infancy, in later time periods could have impact on the risk of skin tumors for susceptible individuals also exposed during infancy. This increase in exposure together with changes in the Ozone layer probably would dilute any effect, if there is one, of month of birth.

In conclusion, we found no association between birth month and later development of skin tumor in a large national cohort study of nearly six million individuals with complete information on exposure, outcome and person‐time at risk.

## CONFLICT OF INTEREST

None declared.

## DISCLOSURE STATEMENT

RL is Professor of epidemiology at the Swedish Medical Products Agency, SE‐751 03 Uppsala, Sweden. The views expressed in this paper do not necessarily represent the views of the Government agency.

## AUTHOR CONTRIBUTION

All authors contributed to design, interpretation of data, and read, revised and approved the final version. MT analyzed data. RL drafted the manuscript.

## ETHICAL APPROVAL

The study was approved by the Regional Ethical Review Board in Stockholm, Sweden (2011/634‐31/4, 2014/417‐32).

## Data Availability

The data that support the findings of this study are available from The National Board of Health and Welfare, Sweden, but restrictions apply to the availability of these data, which were used under license for the current study, and so are not publicly available. Analyses of data are however available from the authors upon reasonable request.
